# Effects and safety of Ginkgo biloba on blood metabolism in type 2 diabetes mellitus: a systematic review and meta-analysis

**DOI:** 10.3389/fendo.2023.1231053

**Published:** 2024-01-08

**Authors:** Huimin Zou, Jingxian Fang, Yu Han, Xue Hu, Jian Meng, Fang Huang, Hui Xu, Chengfei Lu, Yiwen Wang, Lili Zhang, Xiaohong Dong, Yanmei Yu, Yu Guo, Qing Gu, Suijun Wang

**Affiliations:** Department of Endocrinology, Shidong Hospital, Yangpu District, Shidong Hospital Affiliated to University of Shanghai for Science and Technology, Shanghai, China

**Keywords:** Ginkgo biloba, GKB, type 2 diabetes mellitus, T2DM, meta-analysis

## Abstract

**Background:**

There has existed controversy regarding the use of Ginkgo biloba (GKB) for blood metabolism among type 2 diabetes mellitus(T2DM) patients, and we tried to analyze the effects and safety of GKB on T2DM patients.

**Methods:**

We conducted a literature search between January 2003 and December 2022 of seven online databases (PubMed, Scopus, Embase, Google Scholar, Web of Sciences, Cochrane Library, and China National Knowledge Infrastructure). A systematic literature review and meta-analysis were performed to compare the effects and safety of GKB among T2DM patients. Four groups of parameters were extracted and analyzed: hemorheology parameters, lipid profile, glycemic control markers, and adverse events.

**Results:**

In the end, 13 eligible articles with 11 indicators among 1573 patients were included. In the hemorheology parameters section, GKB showed significantly lower plasma viscosity (PV) (SMD=-0.91, 95%CI [-1.45, -0.36], *P*<0.01) and hematocrit (Hct) (SMD=-0.60, 95%CI [-0.97, -0.24], *P*<0.01) than the control group. GKB shoed higher velocity of the dorsalis pedis artery (VDPA) (SMD=0.51, 95%CI [0.26, 0.76], *P*<0.01) and ankle brachial index (ABI) (SMD=0.71, 95%CI [0.32, 1.10], *P*<0.01) than the control. In both the lipid profile and glycemic control markers sections, we did not find any difference between GKB and control groups, including total cholesterol (TC), triglyceride (TG), low-density lipoprotein (LDL), high-density lipoprotein (HDL), hemoglobin A1c (HbA1c), and fasting serum glucose (FSG). In addition, we saw no difference in adverse events (AE). The sensitivity analysis and funnel plot showed that the results in this research were robust and had no publication bias.

**Conclusion:**

In conclusion, GKB might safely reduce the risk of peripheral arterial or even systemic cardiovascular disease. However, GKB did not directly improve lipid and blood glucose levels in T2DM patients.

**Systematic review registration:**

https://inplasy.com/, identifier INPLASY202350096.

## Introduction

Diabetes has become a top global health problem since its incidence has substantially expanded over the past few decades in both industrialized and developing nations (1, 2). In 2021, an estimated 537 million persons (aged 20 to 79) had diabetes, and by 2045, that number was projected to rise to 783 million according to research by the World Health Organization (WHO) and Global Burden of Disease (GBD) (3).

A serious concern for healthcare was diabetes mellitus (DM), which has several features including chronic hyperglycemia and aberrant protein, fat, and carbohydrate metabolism (2, 4–6). Diabetes is one of the five priority noncommunicable diseases (NCDs) in WHO’s NCDs control strategy (7, 8). Ongoing complex, expensive efforts are required to prevent and treat chronic problems in patients with diabetes, especially type 2 diabetes mellitus (T2DM). Peripheral arterial disease (PAD) was one of the most prevalent long-term consequences of T2DM, particularly in elderly patients (5).

Ginkgo biloba (GKB), a deciduous tree of the Ginkgo family, is sometimes referred to as a “living fossil” since it is the oldest gymnosperm that remains after the quaternary glaciation. The pharmacological effects of GKB included antioxidant, anticancer, hepatoprotective, and cardiovascular protection (9). GKB is used to treat metabolism disorders and is frequently classified as a “safe dietary supplement” that can be sold without restriction in pharmacies and health food shops all over the world (4). Several medications for T2DM have had an unsatisfactory effect because of their high costs, unclear safety, side effects, low drug tolerance, and drug resistance. In this situation, phytotherapy has gained a lot of popularity as a viable alternative to pharmacological therapy (4).

There is still controversy about the clinical effects of GKB on blood metabolism in patients with T2DM. Some studies have suggested that GKB was beneficial in improving T2DM-related complications (2, 7, 10), but others found that the efficacy of GKB on diabetic macrovascular complications was not yet significant (11). Therefore, in this study, we analyzed the effects of ginkgo on blood metabolism in diabetic patients based on a large sample with comprehensive parameters.

## Methods

### Literature search strategy

This research was conducted based on the Preferred Reporting Items for Systematic reviews and Meta-Analyses (PRISMA) flow diagram and checklist, which was registered at the International Platform of Registered Systematic Review and Meta-analysis Protocols (INPLASY202350096). Yu Han and Xue Hu searched seven online databases (PubMed, Embase, Scopus, Web of Sciences, Google Scholar, Cochrane Library, and China National Knowledge Infrastructure) for all randomized controlled trials reporting blood tests of GKB on T2DM patients. After screening literature published between January 2003 and December 2022, 13 articles were included without any language restrictions.

The medical subject terms and keywords used for the search were “Ginkgo biloba”, “G. biloba”, “GKB”, “Type 2 diabetes mellitus”, and “T2DM” in the Boolean expression: [(Ginkgo biloba) OR (G. biloba) OR (GKB)] AND [(Type 2 diabetes mellitus) OR (T2DM)]. Related articles from the references of included research were also searched. Studies were eligible if they contained the following information: random control trials or observational studies reporting blood metabolism: hematological parameters, lipid profile, glycemic control markers, and adverse events.

Jian Meng and Fang Huang screened the titles, abstracts, and full text of identified articles during the search and evaluated the risk of bias, extracting the data using a standardized data form. When there was any discrepancy, it was resolved by discussion with a third author (Hui Xu).

### Inclusion and exclusion criteria

After the primary selection of the studies, the texts of the studies that were potentially relevant were reviewed, and the studies were required to meet the following inclusion criteria:

(1) Research should be on the comparison between GKB and control in T2DM patients;
(2) The design of the research should be randomized control trials (RCTs);
(3) Studies should contain blood metabolism indicators evaluating the efficacy and safety of GKB;
(4) The full text of the studies should be available.

Studies were excluded based on the following pre-determined exclusion criteria:

(1) The study contains research on other drugs;
(2) The study contains other topics about T2DM;
(3) The study lacks available data;
(4) The study type is review or abstract.

### Data extraction and quality assessment

From each published study, two writers (Minhui Zou and Jingxian Fang) retrieved the following data: the name of the initial author, publication year, author’s nationality, groups based on intervention (GKB and placebo), research design, sex, average age, and research cycle. The outcome items were blood metabolism parameters.

The quality of the included studies was evaluated by two independent reviewers (Qing Gu and Suijun Wang) using the ROB 2.0 scale (a revised Cochrane risk-of-bias tool for randomized trials).

### Statistical analysis

Using the Cochrane Q statistic and Higgins and Thompsons’ *I²*, we evaluated heterogeneity. *I²* was used to categorize heterogeneity as low, moderate, or high depending on its value: 25%, 50%, or 75%. The fixed-effect model was utilized for the meta-analyses when *I²* was less than 50%. Otherwise, the random-effect model was adopted if *I²* was greater than 50%. More specifically, data for standard mean difference (SMD) with a 95% confidence interval (CI) were retrieved or recalculated for effect sizes of continuous outcomes. For categorical results, odds ratios (ORs) were employed. R version 4.2.1 (R Core Team, Vienna, Austria) and the R package meta (version 6.2.0) were used for statistical analyses. A *P value* lower than 0.05 was considered statistically significant for all reported *P value*s.

Using Egger’s regression test and a funnel plot, publication bias was evaluated for the primary outcome of blood metabolism. We performed a sensitivity analysis to evaluate the robustness of the final results by removing one included article in turns.

## Results

### Search process

**Figure 1:**
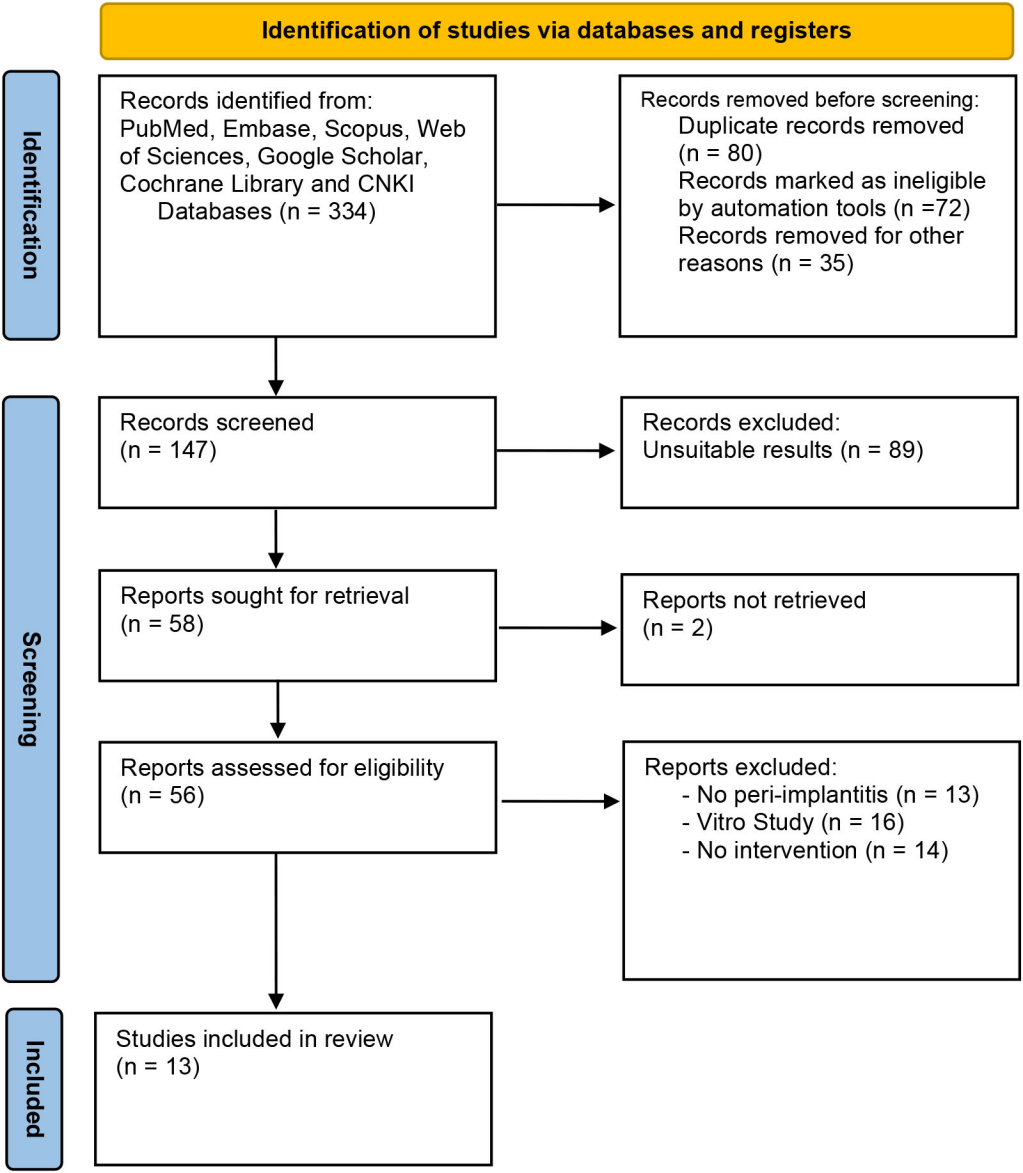
PRISMA diagram of data selection.

Based on the PRISMA flow diagram and inclusion/exclusion criteria, the systematic search of seven online databases identified 334 articles. After duplicate exclusion and screening for eligible research, 13 articles with 1573 patients were suitable for this meta-analysis (12–24). **Figure 1** shows the flow chart of research selection, and the characteristics of the included studies are shown in **Table 1**. The risk of bias assessment for each included study is shown in **Supplementary Files 1**, **2**.

**Table 1:** Main characteristics of the studies included.

Study	Year	Language	Country	Groups	Intervention details	No.of patients (male/female)	Age range (mean)	n	Study design	Years of follow-up
**Aziz1**	2018	English	Iraq	GKB	Ginkgo biloba extract +metformin	5/22	48.7 ± 9.6	27	RCT	December 2016 and October 2017
				Controls	placebo +metformin	3/17	48.2 ± 10.3	20		
**Aziz2**	2018	English	Iraq	GKB	Ginkgo biloba extract +metformin	6/16	50.7 ± 7.8	22	RCT	December 2016 and October 2017
				Controls	placebo+metformin	2/16	47.3 ± 10.8	18		
**Li**	2003	Chinese	China	GKB	Ginkgo Leaf Extract	26/6	68.8 ± 8.56	32	RCT	–
				Controls	Buflomedil	18/5	68.1 ± 8.53	23		
**Li**	2007	Chinese	China	GKB	Ginkgo Leaf Extract+metformin	19/15	66.9 ± 7.3	34	RCT	June 2003 and June 2005
				Controls	metformin	15/14	68.2 ± 7.7	29		
**Liu**	2013	Chinese	China	GKB	Ginkgo Leaf Extract+metformin	–	–	25	RCT	January 2010 and October 2012
				Controls	metformin	–	–	25		
**Liu**	2015	Chinese	China	GKB	Ginkgo Leaf Extract+metformin	59/44	53.2 ± 11.7	103	RCT	January 2014 and November 2014
				Controls	metformin	63/44	57.3 ± 6.9	107		
**Pan**	2007	Chinese	China	GKB	Ginkgo Leaf Extract+metformin	–	57.61 ± 11.78	35	RCT	–
				Controls	metformin	–	56.3 ± 10.92	33		
**Shi**	2019	English	China	GKB	Ginkgo biloba Tablets +Liuwei Dihuang Pills	146/154	60.81 ± 6.36	300	RCT	–
				Controls	placebos	151/149	60.45 ± 6.19	300		
**Sun**	2013	Chinese	China	GKB	Ginkgo Leaf Extract+metformin	16/10	52.4 ± 7.6	26	RCT	January 2011 and December 2012
				Controls	metformin	11/13	55.8 ± 9.1	24		
**Wang**	2020	Chinese	China	GKB	Ginkgo Leaf Extract+metformin	22/25	63.97 ± 1.96	47	RCT	March 2017 and February 2019
				Controls	metformin	26/21	64.11 ± 1.38	47		
**Wu**	2007	Chinese	China	GKB	Ginkgo Leaf Extract+metformin	30/20	61.4 ± 3.7	50	RCT	2004 and 2006
				Controls	metformin	32/16	60.8 ± 3.2	48		
**Xiao**	2016	Chinese	China	GKB	Ginkgo Leaf Extract+metformin	20/25	50.45 ± 4.85	45	RCT	January 2014 and June 2015
				Controls	metformin	19/26	50.65 ± 4.75	45		
**Xie**	2022	Chinese	China	GKB	Ginkgo biloba Tablets+metformin	37/17	63.4 ± 13.72	54	RCT	January 2018 and December 2018
				Controls	metformin	31/23	64.11 ± 1.38	54		

“-” means 'N/A'.

The overall quality of the included studies based on ROB 2.0 was found to be adequate with regard to selection bias, comparability quality, and outcome quality.

### Characteristics of included studies

Of all studies, 3 articles included >100 patients and 10 studies included <100 patients. The research period ranged from 1 to 3 years. The study design of all the included articles was RCTs. The mean age ranged from 47.3 to 68.8 among the included articles. The total number of males and females was close. The detailed characteristics of the included papers are shown in **Table 1**.

### Results of quality assessment

The ROB 2.0 summary and details are shown in **Supplementary Files 1**, **2**, respectively. In summary, less than 20% of articles showed a high risk of bias while over 50% showed a low risk of bias. The details showed that only two articles had an overall high risk of bias and seven had an overall low risk of bias.

### Results of heterogeneity test

**Figure 2:**
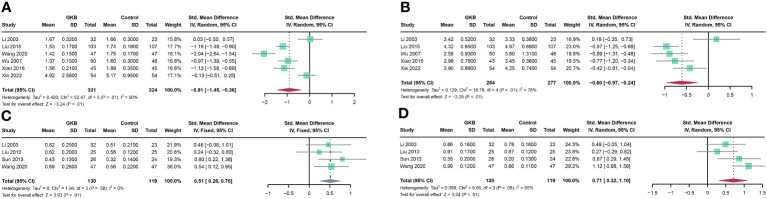
Effects of Ginkgo biloba on hematological parameters of T2DM patients maintained treated with GKB extract and placebo. **(A)** Plasma viscosity (mPa/s). **(B)** Hematocrit (%). **(C)** Velocity of dorsalis pedis artery (m/s). **(D)** Ankle brachial index. SD, standard deviation; 95% CI, 95% confidence interval; Ch*I²*, chi-squared test; Tau2, Tau-squared; *I²*, I-squared; *P*, probability.

Firstly, we analyzed the hemorheology parameters. Concerning plasma viscosity (PV), pooled estimates of six studies showed that GKB had significantly lower PV than the control group (SMD=-0.91, 95%CI [-1.45, -0.36], *P*<0.01) (**Figure 2A**). Five articles reported hematocrit (Hct), and meta-analysis results showed that GKB also had lower Hct than control (SMD=-0.60, 95%CI [-0.97, -0.24], *P*<0.01) (**Figure 2B**). In terms of velocity of dorsalis pedis artery (VDPA) analysis, a meta-analysis of four articles indicated that GKB had higher VDPA than the control (SMD=0.51, 95%CI [0.26, 0.76], *P*<0.01) (**Figure 2C**). The pooled result of the ankle brachial index (ABI) in GKB was higher than the control (SMD=0.71, 95%CI [0.32, 1.10], *P*<0.01) (**Figure 2D**).

**Figure 3:**
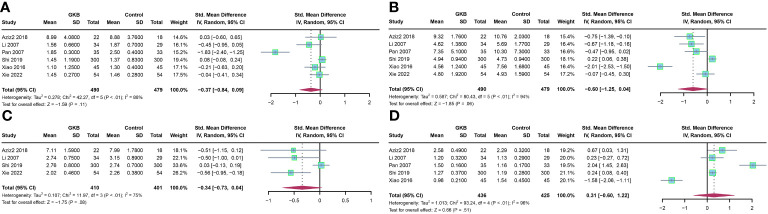
Effects of Ginkgo biloba on lipid profile of T2DM patients maintained treated with GKB extract and placebo. **(A)** Total cholesterol (mmol/L). **(B)** Triglyceride (mmol/L). **(C)** Low-density lipoprotein (mmol/L). **(D)** High-density lipoprotein(mmol/L).

Furthermore, we analyzed the lipid profile after treatment of GKB. Upon total cholesterol (TC)(mmol/L) comparison, GKB showed no difference compared to the control (SMD=-0.37, 95%CI [-0.84, 0.09], *P*=0.11) (**Figure 3A**). Pooled analysis of triglyceride (TG) (mmol/L) showed no difference between GKB and control (SMD=-0.60, 95%CI [-1.25, 0.04], *P*=0.06) (**Figure 3B**). Both low-density lipoprotein (LDL) (mmol/L) (SMD=-0.34, 95%CI [-0.73, 0.04], *P*=0.08) (**Figure 3C**) and high-density lipoprotein (HDL) (mmol/L) (SMD=0.31, 95%CI [-0.60, 1.22], *P*=0.51) (**Figure 3D**) showed no difference compared to the GKB and control groups.

**Figure 4:**
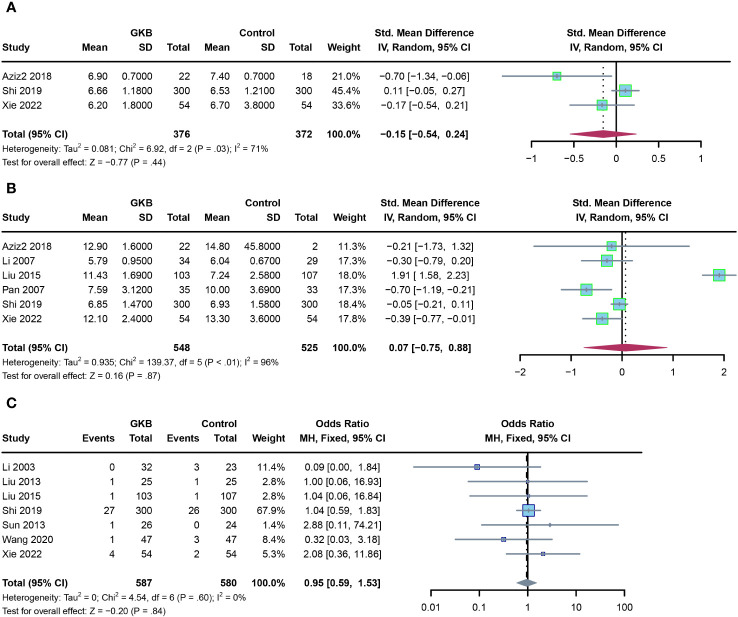
Effects of Ginkgo biloba on glycemic control markers of T2DM patients maintained treated with GKB extract and placebo, and adverse events. **(A)** Hemoglobin A1c (%). **(B)** Fasting serum glucose (mmol/L). **(C)** Adverse events.

Finally, we evaluated the glycemic control markers and adverse events in the GKB and control groups. Meta-analysis of both hemoglobin A1c (HbA1c)(%) (SMD=-0.15, 95%CI [-0.54, 0.24] *P*=0.44) (**Figure 4A**) and fasting serum glucose (FSG)(mmol/L) (SMD=0.07, 95%CI [-0.75, 0.88] *P*=0.87) (**Figure 4B**) showed no difference between the GKB and control. There was also no difference between the two groups in adverse events (AE) (OR=0.95, 95%CI [0.59, 1.53], *P*=0.84) (**Figure 4C**).

### Results of sensitivity analysis and publication bias

**Figure 5:**
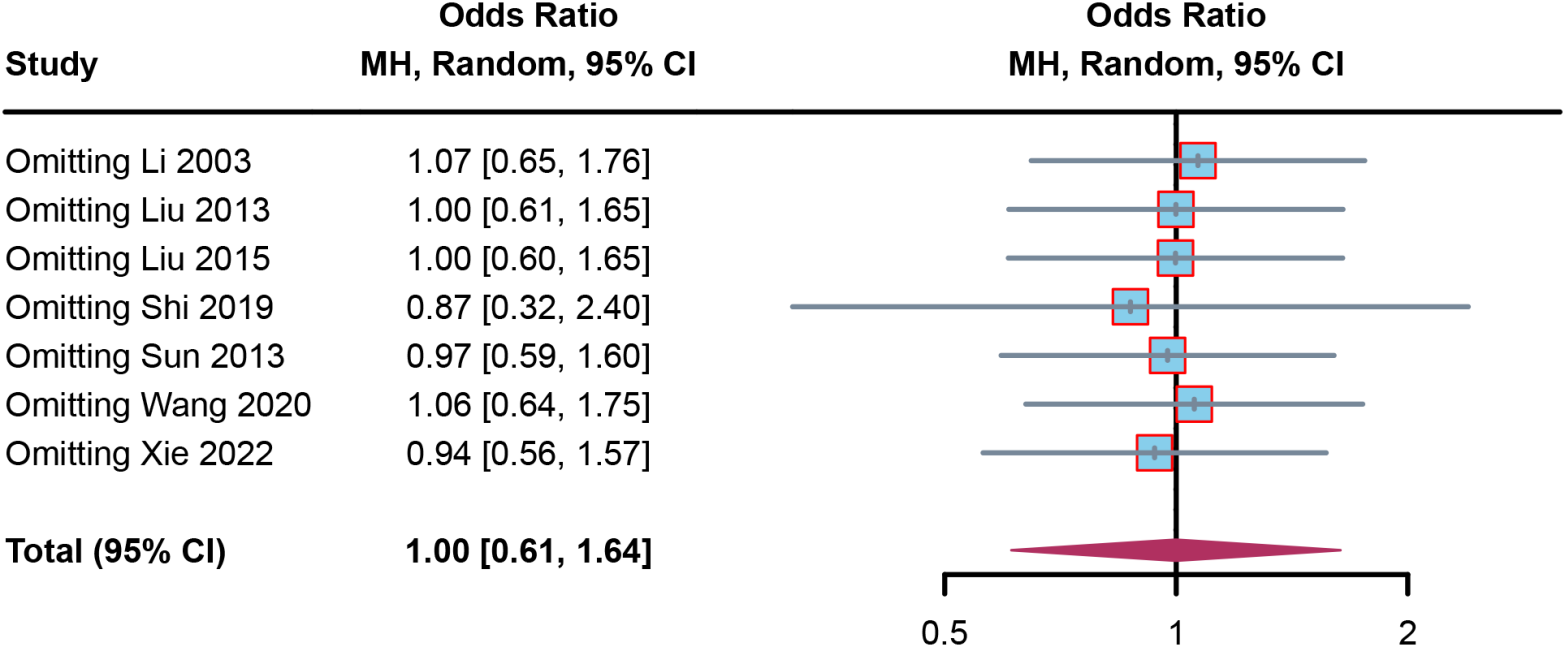
Funnel plot for potential publication bias.

To confirm the robustness of the results, sensitivity analysis was performed. In the sensitivity forest plot (**Figure 5**), we omitted each article in turn. In the adverse events meta-analysis, each included article had a similar OR for robustness; Li (2003) had the highest OR, which was a value of 1.07 [0.65, 1.76], while Shi (2019) had the lowest OR, which was a value of 0.87 [0.32, 2.40]. These results indicated the results were robust.

**Figure 6:**
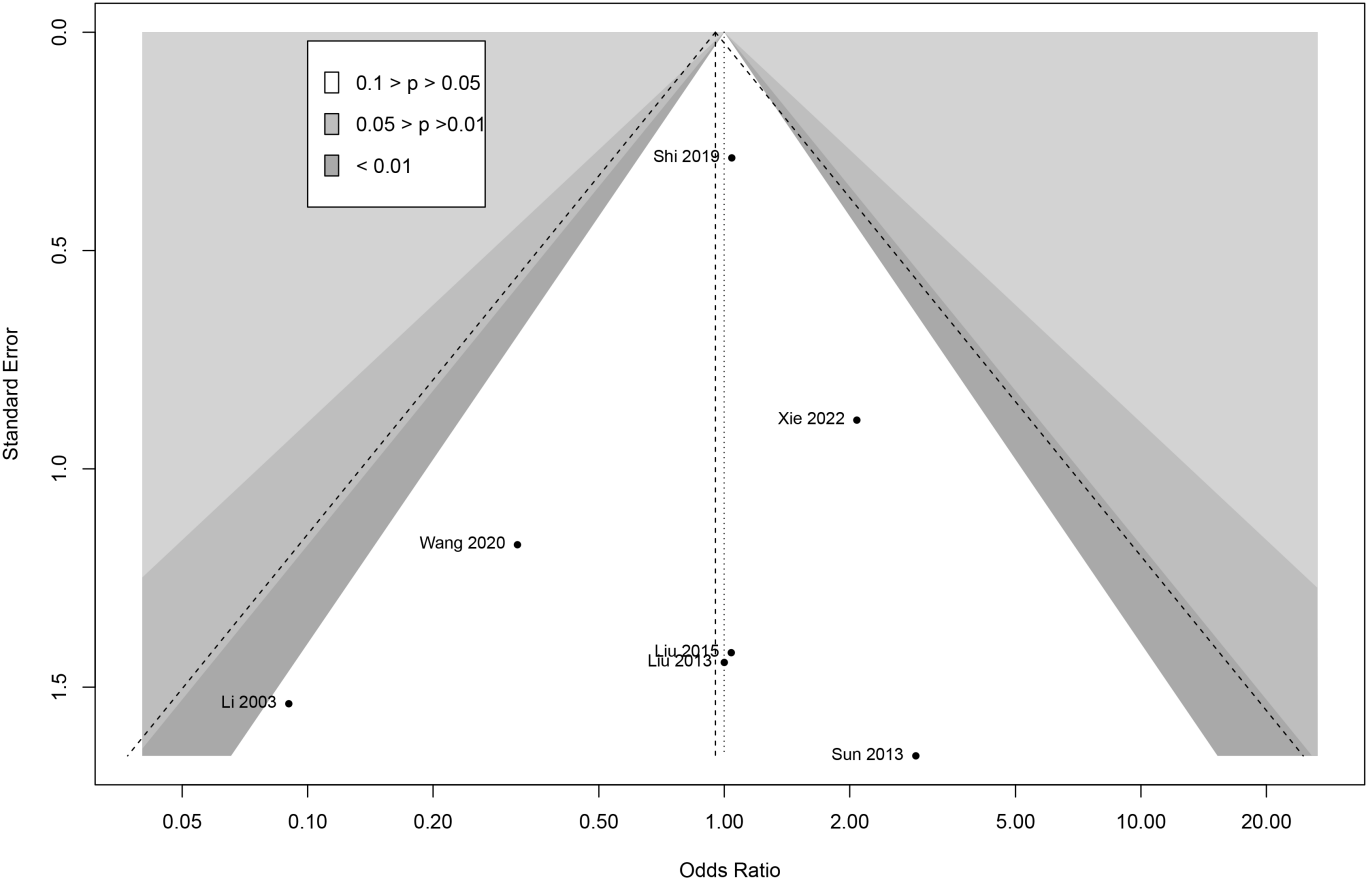
Sensitivity analysis by omitting article in turn.

To analyze the publication bias, Begg funnel plots were performed for adverse events meta-analysis. The funnel plots showed visual symmetry and indicated that limited publication bias existed in this research (**Figure 6**). Egger regression was used to test for adverse events (z = 0.24; *P* = 0.21), which further demonstrated the absence of publication bias.

## Discussion

To our knowledge, this research is the first comprehensive report of GKB on blood metabolism in T2DM with multiple parameters. GKB significantly reduced PV and Hct but increased VDPA and ABI in T2DM patients. In addition, GKB had no effects on TC, TG, LDL, HDL, HbA1c, and FSG. Furthermore, GKB was safe for T2DM patients.

All four significant indicators were associated with PAD in T2DM, and the viscosity of blood was an indicator for understanding and treating the disease (25). In a cross-sectional study in Edinburgh, Lowe et al. found that blood viscosity and its major determinants (Hct, PV, and fibrinogen) as well as leukocyte activation were significantly associated with an increase in the severity of PAD (26). Tzoulaki et al., also in the Edinburgh Arterial Prospective Study, found that the effect of blood viscosity on PAD was modest. Elevated levels of Hct maintained a significant correlation with PAD and showed a dose–response relationship with disease severity (27). Indeed, GKB could lower PV and Hct, which further decreased the risk of PAD. The mechanism by which higher Hct affects coronary blood flow velocity may be that as the hematocrit rises, blood viscosity increases, enhancing the shear force on the vessel wall. This results in damage to the vascular endothelium, which stimulates the release of multiple vasoactive factors from endothelial cells, resulting in an imbalance of vasodilator/constrictor factors (e.g., an imbalance in the secretion of nitric oxide/endothelin), which contributes to the development of PAD (28).

There is a higher prevalence of lesions in the dorsalis pedis artery than in popliteal or anterior/posterior tibial arteries; the dorsalis pedis artery was the first artery to develop diabetic macrovascular disease and was crucial for early screening (29). It could be viewed as a representation of the systemic vasculature in T2DM. The ABI consists of serial measurements of Doppler-recorded systolic blood pressure in the brachial, dorsalis pedis, and posterior tibial arteries. There is concordance between the VDPA and the ABI (30). The sensitivity and specificity of the ABI for the identification of PAD are greater than 90%. There is a well-known relationship between PAD and the number of diseased coronary vessels. The ABI may be a surrogate marker for systemic atherosclerosis. Low ABI is associated with a high prevalence of cardiovascular risk factors (31).

In conclusion, all four significant indices could assess PAD in T2DM, and the reduction of PV and Hct with an increase of VDPA and ABI might reduce the risk of PAD or even systemic vascular disease. However, there was no discernible difference between the GKB group and the control group in the study’s assessment of lipid and glucose indices, suggesting that GKB might not directly lower lipids and glucose in T2DM patients.

Previous articles reported that the pathogenesis of diabetes mellitus was similar to that of “thirst” in Chinese medicine (mainly showed ‘Yin’ deficiency and blood stasis), and Ginkgo biloba could nourish ‘Yin’ and activate blood (10, 11). It was suggested that GKB could improve insulin resistance and inflammation, reduce the serine phosphorylation of IRS-1 receptor, and decrease NFκB/JNK activation, thereby reducing the release of inflammatory adipokines (32). Besides, diabetes was associated with multiple inflammatory responses, including accumulation of AGEs, leukocyte infiltration, ECM deposition, and cytokine and adhesion molecule expression (32). sRAGE was described as the “sponge” of AGE, which counteracted the deleterious effects of cellular RAGE by binding to serum AGE and AGE-*P*. Studies showed that lower sRAGE levels were associated with a higher risk of T2DM, coronary heart disease, and all-cause mortality (33). Ginkgo was reported to reduce the relative total superoxide dismutase activity in patients with T2DM, leading to higher sRAGE levels and reducing the risk of T2DM (34).

In our results, GKB had no effects on HbA1c. Among the included articles, Aziz (13) and Xie (24) said GKB could decrease HbA1c with a limited sample size. However, Shi (19) reported that GKB did not influence HbA1c with a large sample size. In addition, the research period of Shi was 24 months, whereas Aziz’s was 3 months and Xie’s was 12 months. This situation seems controversial and has led to a hypothesis: if Aziz and Xie enlarge their sample size and study period, will the effects of GKB in T2DM be stable? This topic needs further analysis in the form of future large-scale, multicenter, randomized, double-blind, placebo-controlled trials.

GKB was thought to have the following properties: being cardioprotective and anti-apoptotic, having an anti-platelet activating factor, being an antioxidant, exhibiting free radical scavenging, being neuroprotective, and showing membrane stabilization. By decreasing the transcription of pro-apoptotic caspase-9 and JNK and increasing the activity of anti-apoptotic Bcl-2 protein, Ginkgo biloba extract EGb761 attenuated pro-inflammatory and pro-apoptotic processes, thus preventing the initiation of apoptotic signaling cascade and thus protecting the integrity of the mitochondrial membrane (35). Research has demonstrated that the administration of Ginkgo biloba extract can impede adipogenesis and modulate lipid metabolism, resulting in a decrease in body weight and food consumption (8).

Diabetic peripheral vasculopathy had a prevalence of about 20% in diabetic patients and was a common vascular complication of T2DM, with the lower limb arteries being the main site of onset (36, 37). Therefore, for patients with T2DM, in addition to glucose and lipid control, vascular protection was also important. About the vascular protection mechanism, in a study by Tsai et al, they found that GKB increased Krüppel-Like Factor 2 expression, which then increased endothelial NO synthase (eNOS) expression and NO production (38). GKB increased eNOS in a dose-dependent manner at both the RNA and protein levels. NO is an important mediator of endothelial cell function, inhibiting leukocyte adhesion and migration and preventing platelet aggregation. NO exerts its vascular protective effects by using diastolic smooth muscle cells to reduce vascular tone (39, 40).

Concerning the heterogeneity in part of our results, we have found several possible reasons. Firstly, the different publishing years of the included articles contributed to the heterogeneity. Secondly, the different dosages of GKB might influenced the heterogeneity. Thirdly, the different research measurements and staff might lead to heterogeneity. Fourthly, even though most of the included research was conducted in China, the different sampling cities might be related to heterogeneity since China had a large country scope with different lifestyle and eating habits. Finally, the age and sex distribution in different research varied a lot; the lowest average age was 47.3 ± 10.8 while the highest was 68.8 ± 8.56, while the lowest sex ratio (male/female) was 0.13 but the highest was 4.33. In this situation, we still want to conduct a comprehensive meta-analysis for GKB among T2DM and hope it can lay the foundation for better research in the future.

Overall, the results of this study, based on a large sample and multiple indicators, confirmed that GKB might safely reduce the risk of PAD or even systemic cardiovascular disease in T2DM patients but did not directly improve lipid and blood glucose levels in T2DM patients.

The present study had some limitations. First, because studies of GKB affecting patients with T2DM were not retrieved in the United States or Europe, comprehensive results for multiple populations were lacking. Second, due to the variability of indicators reported in the included studies, there was no way to have a larger sample for each indicator. Finally, the related mechanism of included indicators was not complete, which shows the need for future mechanical research.

## Data availability statement

The original contributions presented in the study are included in the article/**Supplementary Material**. Further inquiries can be directed to the corresponding author.

## Author contributions

Conception and design: QG and SW; Administrative support: LZ, XD, YY, and YG; Provision of study materials or patients: HX, CL, and YW; Collection and assembly of data: XH, JM, and FH; Data analysis and interpretation: HZ, JF, and YH; Manuscript writing: All authors. All authors contributed to the article and approved the submitted version.

## Supplementary material

Supplementary File 1 Risk of bias details assessed by ROB 2.0.

Supplementary File 2 Risk of bias summary assessed by ROB 2.0.

## Glossary

**Table:**

T2DM	type 2 diabetes mellitus
DM	Diabetes mellitus
GKB	Ginkgo biloba
PV	plasma viscosity
Hct	hematocrit
VDPA	velocity of dorsalis pedis artery
ABI	ankle-brachial index
TC	total cholesterol
TG	triglyceride
LDL	low-density lipoprotein
HDL	high-density lipoprotein
HbA1c	hemoglobin A1c
FSG	fasting serum glucose
WHO	World Health Organization
GBD	global burden of disease
NCDs	challenges of noncommunicable diseases
PRISMA	referred Reporting Items for Systematic reviews and Meta-Analyses
INPLASY	International Platform of Registered Systematic Review and Meta-analysis Protocols
SMD	mean difference
Ors	Odds ratio
SD	standard deviation
Chi²	chi-squared test
Tau^2^	Tau-squared
I²	I-squared
P	probability
95%CI	95% confidence interval
AGEs	Advanced glycation end products
ECM	extracellular matrix
sRAGE	Soluble receptor for advanced glycation end products
AGE-P	AGE-peptide
eNOS	endothelial NO synthase
PAD	peripheral arterial disease
RCTs	randomized control trails.
