# Supplementary figures and images for: Effects and safety of Ginkgo biloba on blood metabolism in type 2 diabetes mellitus: a systematic review and meta-analysis
Source: Front Endocrinol (Lausanne). 2024 Jan 8;14:1231053. doi: 10.3389/fendo.2023.1231053 (PMC10804948; doi:10.3389/fendo.2023.1231053)

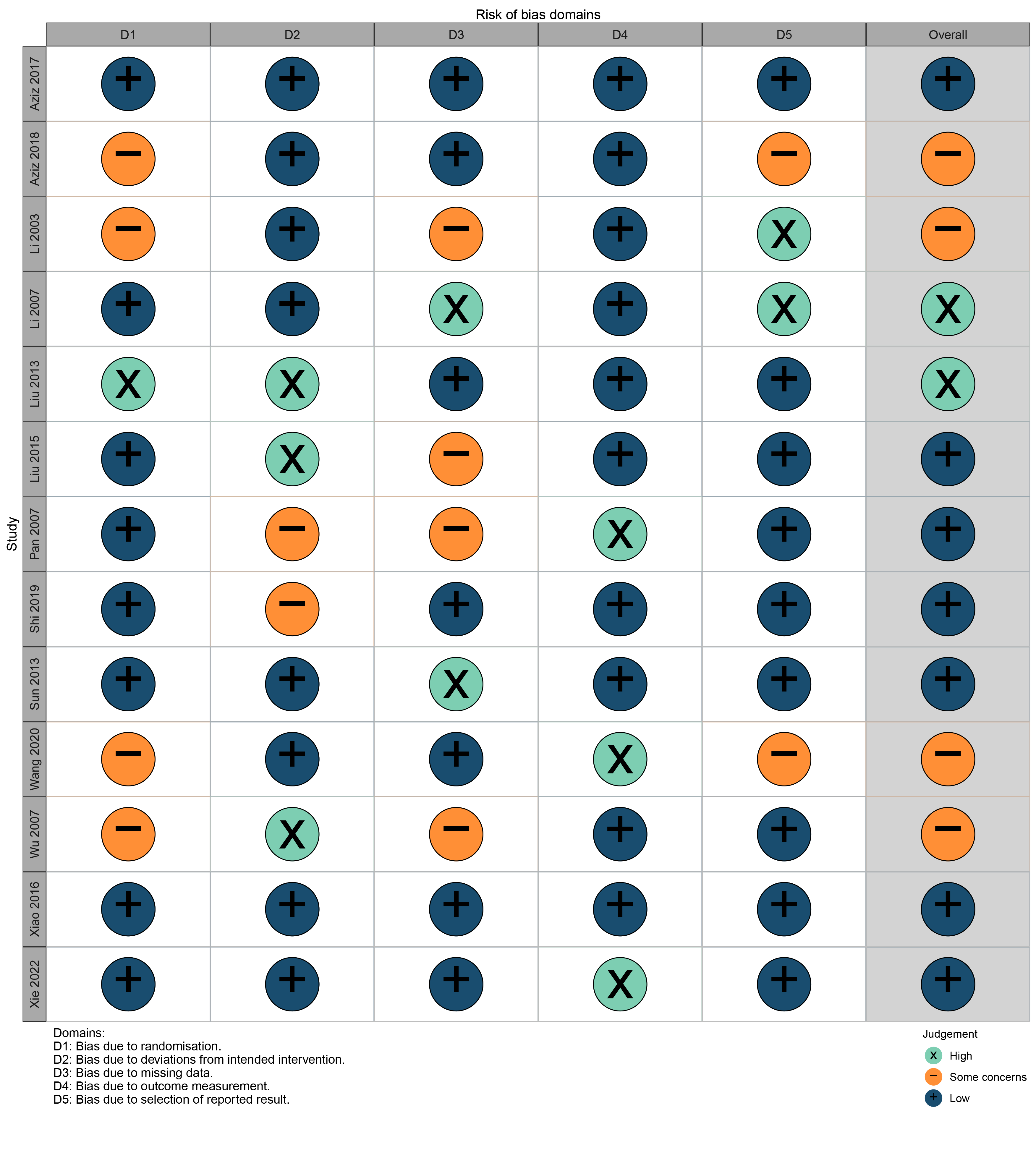

Supplement: Supplementary File 1 — Risk of bias details assessed by ROB 2.0. [file Image_1.tif]

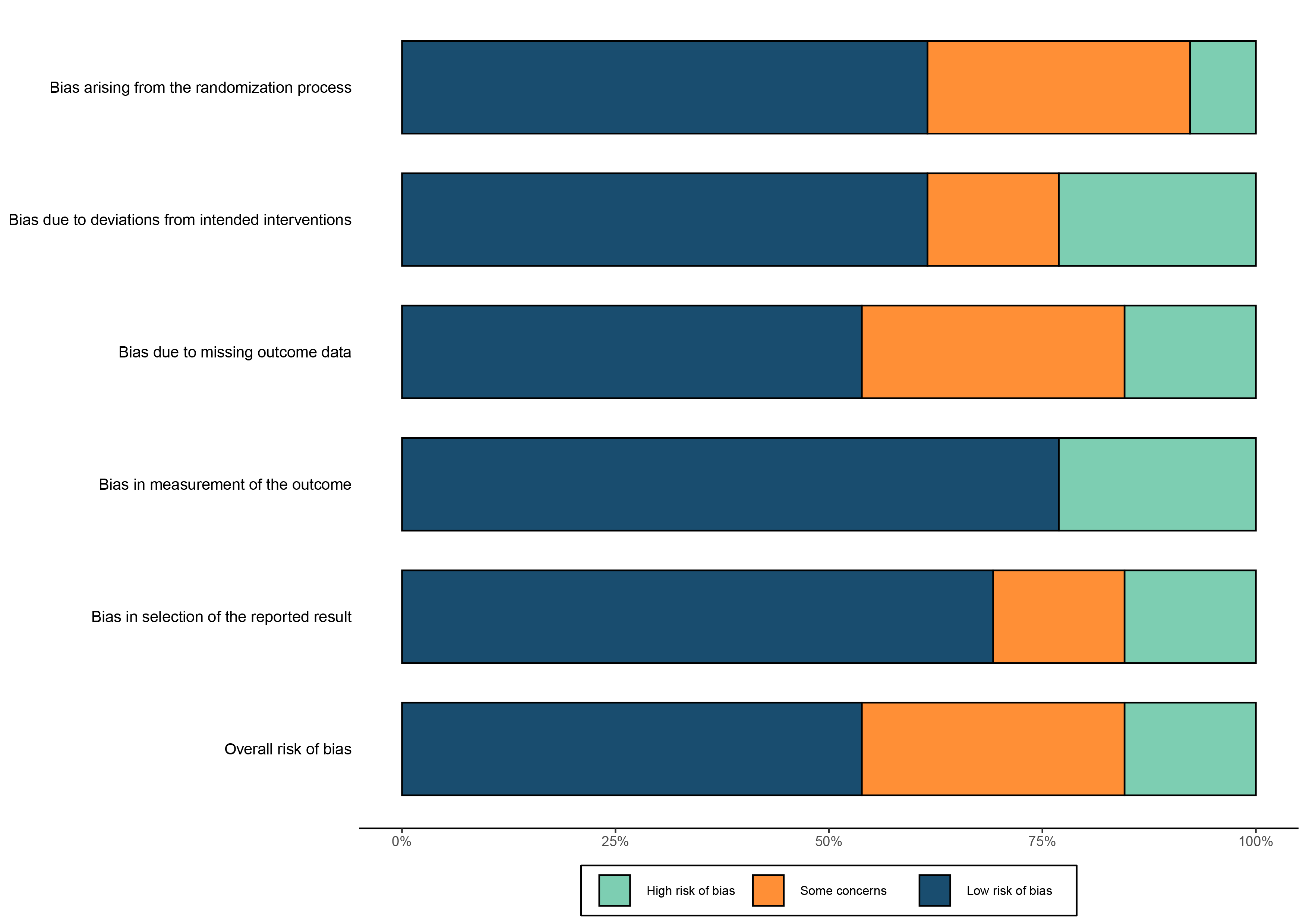

Supplement: Supplementary File 2 — Risk of bias summary assessed by ROB 2.0. [file Image_2.tif]
